# Cerebro-cerebellar gray matter abnormalities associated with cognitive impairment in patients with recent-onset and chronic schizophrenia

**DOI:** 10.1038/s41537-024-00434-8

**Published:** 2024-01-27

**Authors:** Naok Kang, Subin Chung, Sang-Hyuk Lee, Minji Bang

**Affiliations:** 1 Department of Psychiatry, CHA Bundang Medical Center, CHA University School of Medicine, Seongnam, Republic of Korea; 2 CHA University School of Medicine, Pocheon, Republic of Korea

**Keywords:** Schizophrenia, Schizophrenia

## Abstract

Although the role of the cerebellum in schizophrenia has gained attention, its contribution to cognitive impairment remains unclear. We aimed to investigate volumetric alterations in the cerebro-cerebellar gray matter (GM) in patients with recent-onset schizophrenia (ROS) and chronic schizophrenia (CS) compared with healthy controls (HCs). Seventy-two ROS, 43 CS, and 127 HC participants were recruited, and high-resolution T1-weighted structural magnetic resonance images of the brain were acquired. We compared cerebellar GM volumes among the groups using voxel-based morphometry and examined the cerebro-cerebellar GM volumetric correlations in participants with schizophrenia. Exploratory correlation analysis investigated the functional relevance of cerebro-cerebellar GM volume alterations to cognitive function in the schizophrenia group. The ROS and CS participants demonstrated smaller cerebellar GM volumes, particularly in Crus I and II, than HCs. Extracted cerebellar GM volumes demonstrated significant positive correlations with the cerebral GM volume in the fronto-temporo-parietal association areas engaged in higher-order association. The exploratory analysis showed that smaller cerebellar GM in the posterior lobe regions was associated with poorer cognitive performance in participants with schizophrenia. Our study suggests that cerebellar pathogenesis is present in the early stages of schizophrenia and interconnected with structural abnormalities in the cerebral cortex. Integrating the cerebellum into the pathogenesis of schizophrenia will help advance our understanding of the disease and identify novel treatment targets concerning dysfunctional cerebro-cerebellar interactions.

## Introduction

Schizophrenia is a detrimental disorder characterized by structural abnormalities in the brain that result in functional impairments. Although advances in neuroimaging techniques have improved our understanding of schizophrenia^1–3^, its underlying pathogenesis remains unclear. Structural and functional aberrations in the fronto-temporo-limbic areas do not explain the clinical manifestations of schizophrenia across a wide range of mental functions. The cerebellum, traditionally associated with motor coordination, also plays a role as a dynamic coordinator of higher-order cognitive processing through reciprocal connections with cerebral networks^4–6^.

The cerebrum and cerebellum are anatomically and functionally connected through the cortico-cerebello-thalamo-cortical circuit (CCTCC), which comprises closed polysynaptic loops that convey efferents and afferents between them^7^. The multi-scale modular organization of the cerebellum is responsible for modulating cerebral input and returning it to the cerebrum for fine-tuning sensorimotor and cognitive processing^8,9^. Cerebellar lesions, particularly in the posterior lobe, are associated with impaired executive function, spatial cognition, affective flattening, inappropriate behavior, and language deficits that mimic schizophrenia^10,11^. These lesions do not usually cause psychotic symptoms; however, the disruption of the CCTCC is one of the possible mechanisms that explain difficulties in prioritizing, handling, and responding to information that allows coordinated thoughts and behaviors in patients with schizophrenia^12,13^.

Neuroimaging studies of the cerebellum have demonstrated that patients with schizophrenia have smaller cerebellar gray matter (GM) volume than healthy controls (HCs) ^14–16^. These findings have been observed in both antipsychotic-naïve^17^, chronically medicated^18,19^, and first-episode patients with schizophrenia^20–23^. Despite contradictory findings of larger cerebellar GM volumes^24–26^, a multisite mega-analysis of 983 patients has demonstrated that the total cerebellar GM volume is robustly decreased in patients with schizophrenia than that in HCs, and the volume reduction is highly consistent across ages from late adolescence^27^. Furthermore, a smaller cerebellar volume has been observed in unaffected first-degree relatives of patients with schizophrenia^28^ and individuals at ultra-high risk for psychosis^29^, suggesting that structural abnormalities in the cerebellum present before the onset of overt psychosis and may confer susceptibility to schizophrenia. Consistent with the notion that schizophrenia is characterized by poor coordination of cognitive processes^13^, structural changes in the cerebellum have been associated with cognitive impairment across multiple domains in patients with schizophrenia^21,30–32^. Functional neuroimaging studies have also provided compelling evidence of dysfunctional activation of the cerebellum not only during motor tasks, but also during cognitive and affective tasks in patients with schizophrenia^33–38^. However, the relationship between the cerebellum and non-motor higher-order functions remains unclear because the cerebellum intertwines with the cerebrum, and their interaction is highly complicated.

To better understand the role of the cerebellum in the pathogenesis of schizophrenia, an integrative framework of the cerebro-cerebellar interactions through the CCTCC is required. Functional magnetic resonance imaging (MRI) and diffusion tensor imaging (DTI) are common neuroimaging modalities that assess the interactions between distinct brain regions at functional and structural levels, respectively. Previous functional MRI studies have demonstrated that dysfunctional connectivity of the CCTCC is associated with impaired cognitive performance^39,40^ and clinical manifestations, including positive and negative symptoms^41–43^ and self-experience disturbances^44,45^, in patients with schizophrenia. DTI studies have indicated that aberrations in white matter tracts connecting the cerebrum and cerebellum correlate with poor cognitive functioning^46–49^ and clinical symptom severity^50,51^. While both modalities provide useful information on connections between the two parts of the brain, they do not correspond perfectly with each other because functional connectivity is derived from complex polysynaptic interactions in structural networks^52,53^. Furthermore, connectivity analysis does not provide direct information about the characteristics of the GM, which contains neuronal cell bodies and dendritic branches that function to command various mental operations.

Structural GM alterations reflect more stable and enduring changes rather than transient physiological shifts linked to acute psychosis in patients with schizophrenia^54^. Since macroscale brain networks identified based on structural association closely resemble those based on synchronized brain activity rather than white matter architecture^55,56^, investigating cerebellar GM and its associations with the cerebellar cortex would provide additional insights into the cerebro-cerebellar interactions that last throughout the illness course of schizophrenia. Evidence of the cerebro-cerebellar structural association has been found in a clinical observation of crossed cerebellar diaschisis, which refers to a decrease in regional blood flow and metabolism in the contralateral cerebellar hemisphere after unilateral cerebral stroke^57,58^. This phenomenon is consistent with previous findings showing that the cerebellar volume is positively correlated with GM thickness in the corresponding cerebral cortices of patients with schizophrenia^27^ and that functional impairments associated with cerebellar abnormalities are related to their cerebral counterparts^59,60^. Considering that structural abnormalities in one part of the brain could affect another part under shared pathogenic conditions, the association between the cerebral and cerebellar GM structures could facilitate an integrated understanding of the pathogenesis of schizophrenia based on the structural topology.

We aimed to investigate volumetric alterations in the cerebellar GM in patients with recent-onset schizophrenia (ROS) and chronic schizophrenia (CS). We hypothesized the following: (1) the cerebellar GM volume would decrease in patients with ROS and CS than that in HCs, particularly in posterior cerebellar regions; and (2) cerebellar GM volume decrease in patients with ROS and CS would indicate significant correlations with the cerebral GM volume, particularly in regions responsible for non-motor functions, including the fronto-temporo-parietal cortices. Additionally, we aimed to explore the functional relevance of cerebellar abnormalities to cognitive function by conducting a correlation analysis between cerebro-cerebellar GM volume and cognitive performance in participants with schizophrenia.

## Methods

### Participants

We recruited patients with schizophrenia who received psychiatric treatment in either inpatient or outpatient settings at the Department of Psychiatry, CHA Bundang Medical Center (Seongnam, Republic of Korea). HCs were recruited through paper-based and online advertisements from local communities. All participants were Korean, aged 18–65 years. Schizophrenia was diagnosed by experienced psychiatrists based on the Diagnostic and Statistical Manual of Mental Disorders (DSM), Fourth Edition, Text Revision, or DSM-5 criteria. The exclusion criteria were as follows: (1) a history of neurological or neurodevelopmental disorders, head trauma with the loss of consciousness, or other psychiatric comorbidities; (2) clinically significant or unstable medical illness; (3) left-handedness assessed using the Edinburgh Handedness Inventory^61^; and (4) any contraindications for MRI scans. The HCs had no personal or family history of psychiatric disorders. ROS was defined as the presence of overt psychotic symptoms for less than 24 months, with longer durations classified as chronic. Clinical symptoms were assessed using the Positive and Negative Syndrome Scale^62^. Finally, 242 participants (72 patients with ROS, 43 patients with CS, and 127 HCs) were included in this study.

All study procedures were reviewed and approved by the Institutional Review Board of the CHA Bundang Medical Center in accordance with the latest version of the tenets of the Declaration of Helsinki and the principles of Good Clinical Practice. All participants provided written informed consent after receiving a complete explanation of the study procedures.

### Neuroimaging data acquisition and analysis

High-resolution structural brain images were acquired using a 3.0-Tesla GE Signa HDxt scanner (GE Healthcare, Milwaukee, WI, USA) with an eight-channel phase-array head coil. The scan parameters for the T1-weighted three-dimensional fast spoiled gradient-recalled echo sequence were as follows: repetition time = 6.3 ms; echo time = 2.1 ms; flip angle = 12˚; field of view = 256 × 256 mm^2^; matrix = 256 × 256; and voxel size = 1 × 1 × 1 mm^3^. The scanning time was 5 min.

All images were inspected visually for artifacts and distortions, and none of the acquired images were discarded. We analyzed neuroimaging data from the cerebellum using Statistical Parametric Mapping (SPM12; Wellcome Trust Center for Human Neuroimaging, London, UK) and the spatially unbiased intra-tentorial template of the cerebellum and brainstem (SUIT) toolbox (version 3.5; Brain and Mind Institute, Western University, London, Canada)^63^, implemented in MATLAB (version R2021b; MathWorks Inc., Natick, MA, USA). T1-weighted images were preprocessed according to the standard SUIT protocol as follows: (1) alignment of whole-brain images along the anterior-posterior commissure line; (2) isolation of the cerebellum and brainstem; (3) segmentation of the images into tissue-type maps; (4) normalization of these maps into the SUIT atlas template using the diffeomorphic anatomical registration through exponentiated lie algebra algorithm^64^; (5) modulation of normalized cerebellar maps to obtain GM volume by multiplying the Jacobian determinants, derived from the spatial normalization step, to compensate for individual local volume deformations and reslicing; and (6) spatial smoothing of cerebellar GM volume maps with Gaussian kernel of a 4-mm full-width at half-maximum, which was selected based on previous studies to ensure the precise definition of cerebellar structures^31,65–67^. The cerebral images underwent the standard “recon-all” pipeline, implemented in FreeSurfer (version 7.1.0; Athinoula A. Martinos Center for Biomedical Imaging, Charlestown, MA, USA).

### Statistical analysis

All statistical procedures, except for the neuroimaging analysis, were performed using the Statistical Package for the Social Sciences (version 27; IBM Corp., Armonk, NY, USA). Sociodemographic and clinical characteristics of the study participants were compared using analysis of variance and independent *t*-test for continuous variables and chi-squared test for categorical variables.

We performed voxel-based morphometry (VBM) analysis to compare the regional GM volumes of the cerebellum between the ROS, CS, and HC groups using SPM12. Statistical significance was defined as the clusters surviving a voxel-wise threshold of *p* < 0.05 after family-wise error correction for multiple comparisons. The minimum cluster size was set to 100 contiguous voxels. Further, we conducted a post-hoc analysis with the mean volumes extracted from significant cerebellar clusters using analysis of covariance (ANCOVA) and pairwise comparisons with Bonferroni correction. To examine the volumetric correlations between the cerebellar clusters and cerebral GM, we performed a correlation analysis with the mean volumes of each cerebellar cluster using a vertex-wise general linear model in the cerebrum with FreeSurfer. The results were corrected for multiple comparisons with a cluster-forming threshold and cluster-wise threshold of *p* < 0.0001 and *p* < 0.05, respectively. Age, sex, and intracranial volume (ICV) were controlled for as covariates in all statistical analyses of GM volume.

## Results

### Sociodemographic and clinical characteristics

Table 1 summarizes the sociodemographic and clinical characteristics of the participants. The participants with ROS were significantly younger than those with CS or HCs. The CS group had a longer duration of psychosis and a lower proportion of antipsychotic-naïve participants than the ROS group. Since participants with schizophrenia who had been off antipsychotics for at least 6 months were in case of relapse after discontinuation, it was natural for those in the CS group to have a higher proportion of prior exposure to antipsychotics at least 6 months ago than those in the ROS group. Although patients with schizophrenia underwent MRI within 2 weeks of receiving antipsychotics if they exhibited uncooperative behavior due to acutely exacerbated psychotic symptoms, no significant differences were observed between the two groups of schizophrenia in the days of antipsychotics exposure and chlorpromazine equivalent dose of antipsychotics at MRI acquisition. Other characteristics were not significantly different among the groups.

**Table 1:** Demographic and clinical characteristics of the study participants.

	HCs (*n* = 127)	ROS (*n* = 72)	CS (*n* = 43)	Statistics	*p*
Women, *n* (%)	66 (52.0)	50 (69.4)	26 (60.5)	*χ*^2^ = 5.86	0.053
Age (years), mean ± SD	37.8 ± 10.6	33.6 ± 10.6	37.5 ± 12.3	*F* = 3.67	0.027^a^
Intracranial volume (ml), mean ± SD	1524.4 ± 124.1	1493.3 ± 182.1	1482.0 ± 152.6	*F* = 1.79	0.169
Duration of psychosis (months), mean ± SD		4.2 ± 4.5	101.7 ± 94.2	*t* = − 6.78	< 0.001^b^
Exposure to antipsychotics					
Naïve, *n* (%)		68 (94.4)	24 (55.8)	*χ*^2^ = 25.11	< 0.001
>6 months-free, *n* (%)		4 (5.6)	19 (44.2)		
Duration of antipsychotics before MRI acquisition (days), mean ± SD		4.6 ± 3.9	6.2 ± 4.5	*t* = − 1.98	0.050
Dose of antipsychotics (mg/day), mean ± SD^c^		412.3 ± 298.4	506.2 ± 343.8	*t* = −1.54	0.126
PANSS, mean ± SD					
Positive symptoms		29.1 ± 7.0	28.3 ± 7.9	*t* = 0.55	0.581
Negative symptoms		21.4 ± 7.5	22.9 ± 7.2	*t* = − 1.05	0.294
General psychopathology		59.5 ± 13.1	59.8 ± 15.3	*t* = −0.13	0.900

*HCs* healthy controls, *ROS* recent-onset schizophrenia, *CS* chronic schizophrenia, *PANSS* Positive and Negative Syndrome Scale, *SD* standard deviation.

^a^Pairwise comparisons: HCs vs. ROS, *p* = 0.028; HCs vs. CS, *p* > 0.999; ROS vs. CS, *p* = 0.188.

^b^Levene’s test for equality of variances indicated that the variances were not assumed equal between the two groups.

^c^Chlorpromazine equivalent doses were calculated according to Gardner et al.^79^.

### Cerebellar VBM for the Comparison of GM Volume between the ROS, CS, and HC groups

**Fig. 1:**
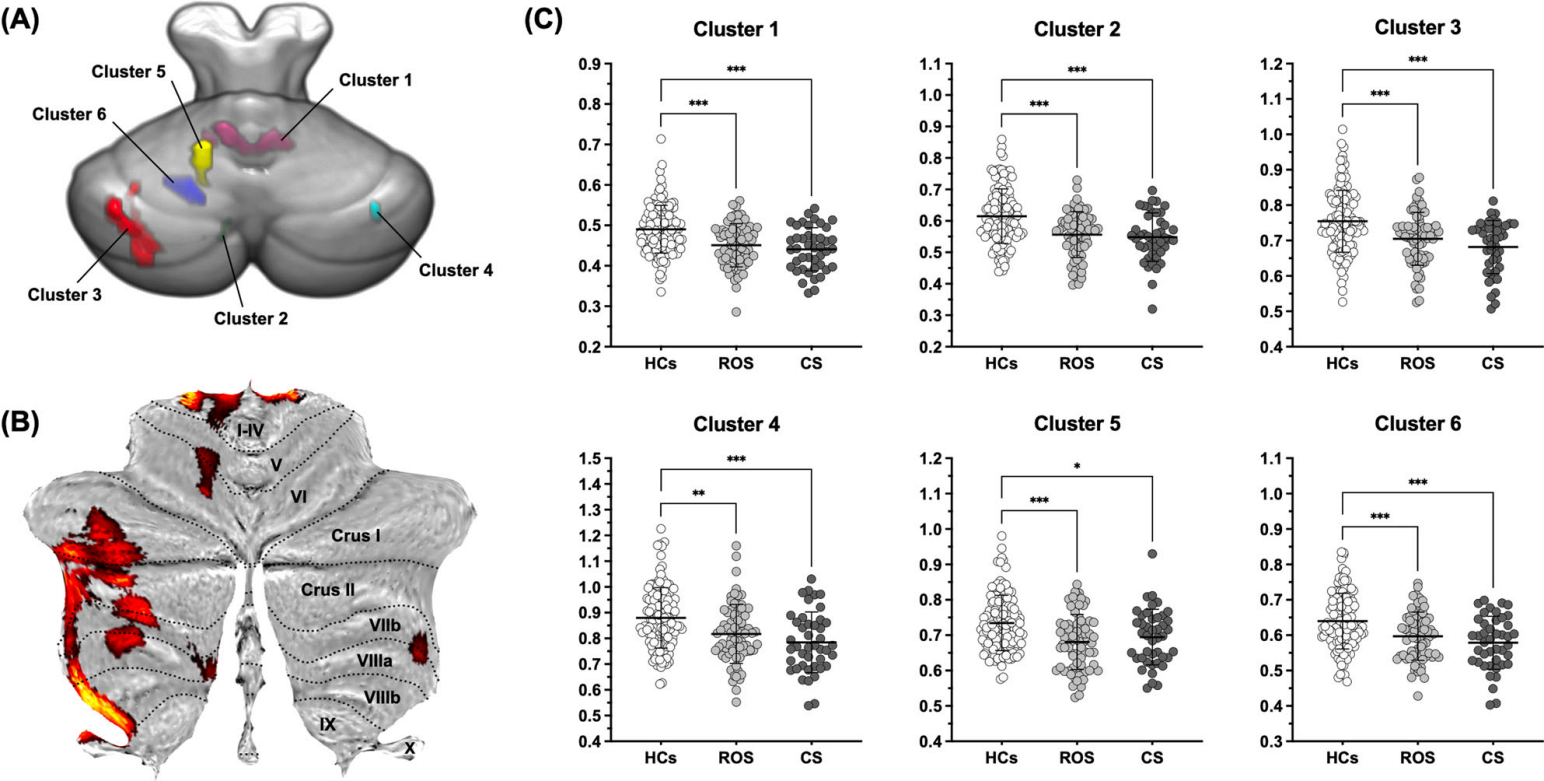
Cerebellar VBM and post-hoc comparisons between the ROS, CS, and HC groups. **A** Results of cerebellar VBM displaying significant between-group differences in GM volume among the ROS, CS, and HC groups (FWE-corrected voxel-wise *p* < 0.05 with a minimum of 100 contiguous voxels). **B** Significant cerebellar clusters visualized on a flat map. **C** Post-hoc pairwise comparisons within significant cerebellar clusters. VBM voxel-based morphometry; GM gray matter; ROS recent-onset schizophrenia; CS chronic schizophrenia; HC healthy control; FWE family-wise error.

Six clusters demonstrating significant differences in cerebellar GM volume were observed between the ROS, CS, and HC groups (Fig. 1A, B). Table 2 presents the cluster information for these cerebellar regions, indicating significant between-group differences in their GM volume. The post-hoc pairwise analysis demonstrated that both schizophrenia groups had smaller GM volumes in all six clusters than the HCs, and no significant differences were observed between the ROS and CS groups (Fig. 1C). The statistical significance of these differences remained unchanged after controlling for the duration of psychosis, pre-study exposure to antipsychotics, and duration and chlorpromazine equivalent dose of antipsychotics at MRI scan.

**Table 2:** Cerebellar clusters showing significant between-group differences in GM volume among the ROS, CS, and HC groups.

	Cerebellar region	MNI coordinate			*F*	*Z*	Cluster size	Cluster-level FWE-corrected *p*
		x	y	z				
Cluster 1	Right lobules I–IV	5	−44	−24	25.51	6.37	922	<0.001
	Left lobules I–IV	−13	−39	−28	20.02	5.62		
	Right lobules I–IV	13	−40	−29	18.64	5.42		
Cluster 2	Left lobule X	−22	−41	−43	19.94	5.61	448	<0.001
Cluster 3	Left Crus I	−43	−56	−42	17.56	5.25	1779	<0.001
	Left Crus II	−33	−63	−44	16.89	5.14		
	Left Crus II	−35	−47	−43	15.95	4.98		
Cluster 4	Right lobule VIIb	39	−46	−50	13.88	4.61	129	0.006
Cluster 5	Left lobule V	−14	−54	−21	13.85	4.61	542	<0.001
	Left lobule VI	−14	−61	−26	13.58	4.56		
Cluster 6	Left lobule VIIIa	−8	−65	−43	13.33	4.51	102	0.009

*GM* gray matter, *ROS* recent-onset schizophrenia, *CS* chronic schizophrenia, *HC* healthy control, *MNI* Montreal Neurological Institute, *FWE* family-wise error.

### Cerebro-cerebellar GM volumetric associations in participants with Schizophrenia

**Fig. 2:**
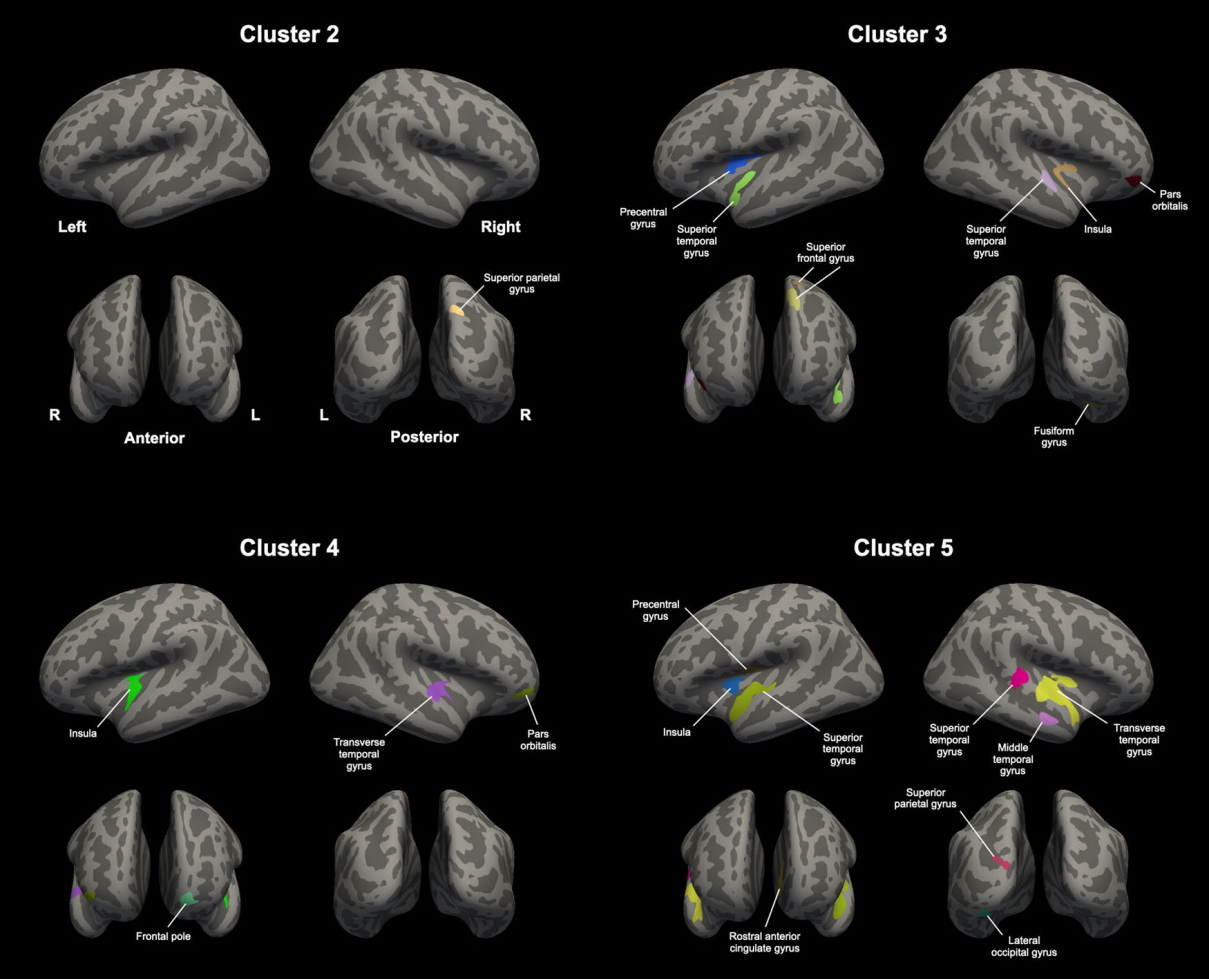
Cerebro-cerebellar gray matter volumetric associations in the participants with schizophrenia (cluster-forming threshold of *p* < 0.0001; cluster-wise threshold of *p* < 0.05).

Cerebellar GM volumes extracted from clusters 2 to 5 were positively correlated with the cerebral GM volumes in the fronto-temporo-parietal association areas (Fig. 2, Table 3). This suggests that the cerebral regions displaying volumetric associations with the cerebellar clusters were distributed in the ipsilateral and contralateral hemispheres. Notably, clusters 1 and 6 did not demonstrate significant cerebro-cerebellar associations.

**Table 3:** Cerebral GM Mvolumetric correlations with the cerebellar clusters in participants with schizophrenia.

Cerebellar cluster	Cerebral region	MNI coordinate			Size (mm^2^)	Vertices	Cluster-wise *p*
		x	y	z			
Cluster 1	N/A						
Cluster 2	Right						
	Superior parietal gyrus	21.6	−71.5	42.3	179.77	304	0.004
Cluster 3	Left						
	Precentral gyrus	−38.9	−3.0	17.3	472.35	1325	<0.001
	Superior frontal gyrus	−6.8	28.5	53.8	489.56	872	<0.001
		−17.2	10.4	60.1	250.27	381	<0.001
	Superior temporal gyrus	−40.5	−18.9	−9.4	344.74	829	<0.001
	Right						
	Fusiform gyrus	30.1	−62.4	−10.0	335.33	610	<0.001
	Pars orbitalis	43.7	43.7	−8.8	269.24	367	0.001
	Insula	36.0	−0.1	6.9	278.20	746	0.001
	Superior temporal gyrus	55.7	−10.5	1.2	236.95	562	0.002
Cluster 4	Left						
	Insula	−38.9	−20.	−7.7	372.40	818	<0.001
	Frontal pole	−10.1	62.0	−8.1	209.36	266	0.002
	Right						
	Transverse temporal gyrus	52.4	−11.9	2.1	462.47	1185	0.002
	Pars orbitalis	40.4	46.0	−4.6	260.30	362	0.002
Cluster 5	Left						
	Superior temporal gyrus	−43.2	−18.5	−9.6	815.64	1812	<0.001
	Rostral anterior cingulate gyrus	−6.6	38.2	0.2	263.48	489	<0.001
	Precentral gyrus	−45.8	−6.8	11.9	242.24	679	0.001
	Insula	−33.5	2.3	6.9	226.67	545	0.001
	Lateral occipital gyrus	−27.5	−87.4	−15.4	211.45	276	0.001
	Superior parietal gyrus	−19.6	−85.9	16.2	185.47	237	0.004
	Right						
	Transverse temporal gyrus	53.4	−10.8	1.4	1357.87	3226	<0.001
	Superior temporal gyrus	65.3	−26.2	5.3	345.22	785	0.002
	Middle temporal gyrus	51.3	−11.4	−21.4	222.76	319	0.003
Cluster 6	N/A						

*GM* gray matter, *MNI* Montreal Neurological Institute, *N/A* not available.

### Exploratory analysis for cognitive performance and its correlations with cerebro-cerebellar GM volumes in participants with Schizophrenia

Cognitive function was evaluated using a comprehensive neurocognitive battery in participants with schizophrenia (Supplementary Material S1). Cognitive function performance between the ROS and CS groups was compared using ANCOVA, with age, sex, ICV, duration of psychosis, and chlorpromazine equivalent dose of antipsychotics as covariates. We observed no significant differences in the neurocognitive performance between the ROS and CS groups, except for delayed recognition of verbal memory (Supplementary Table S1).

The extracted mean GM volumes of the cerebellar clusters were analyzed for correlations with cognitive performance using partial correlation analysis, with age, sex, ICV, duration of psychosis, and chlorpromazine equivalent dose of antipsychotics as covariates. This analysis indicated that the mean GM volumes were associated with cognitive performance in several domains, including executive function and verbal memory (Supplementary Table S2). Notably, greater mean GM volume extracted from the cerebral regions associated with cerebellar clusters 3–5 positively correlated with better performance on cognitive tests for executive function, verbal memory, and visual memory (Supplementary Table S3). Detailed results of the exploratory correlation analysis are presented in Supplementary Material S2. However, none of these correlations remained significant after the Bonferroni correction for multiple comparisons was applied.

## Discussion

Both the ROS and CS groups, compared with HCs, had significantly smaller GM volumes in the cerebellar regions located primarily in the posterior lobe. Notably, no significant GM volume differences were observed between the ROS and CS groups. Furthermore, GM volumes in the posterior cerebellar regions were positively correlated with those in the fronto-temporo-parietal cortices engaged in higher-order cognition. Exploratory correlation analysis with cognitive function showed an incipient trend that cerebellar GM abnormalities in schizophrenia are structurally and functionally related to the cerebrum in terms of cognitive function. Our results support previous findings that the pathogenesis of schizophrenia is shared between the cerebrum and the cerebellum and is evident in the early stages of the illness by replicating previous mega-analytic findings^27^ in an independent sample.

Smaller cerebellar GM volume in patients with schizophrenia than that in HCs is consistent with findings from previous neuroimaging studies^14–23^. The lack of significant differences in cerebellar GM volume between participants with ROS and those with CS corresponds with a previous result showing that cerebellar GM volume differences were stationary across an age range, from adolescence to old age, between patients with schizophrenia and HCs^27^. This suggests that structural abnormalities in the cerebellum may be involved in the early pathogenesis of schizophrenia rather than as a consequence of chronic disease progression. Notably, the regions displaying significant differences between the participants with schizophrenia and HCs, including the lobules I–IV, V, VII, and Crus I and II, overlapped with those with decreased GM volume in a meta-analysis of first-episode antipsychotic-naïve patients^68^. Based on cerebellar parcellation estimated by the intrinsic functional connectivity coupled with the cerebrum, the identified cerebellar regions were affiliated not only with the somatomotor network but also with the fronto-parietal and dorsal/ventral attention networks involved in higher-order cognitive functions^4^. The largest cluster spans Crus I and II, which are the major structures of the cerebellar counterparts of the cerebral fronto-parietal network. A previous mega-analysis also indicated the greatest effect size for volume decreases in cerebellar regions connected to the fronto-parietal network^27^.

Beyond the traditional perspective that the cerebellum is solely involved in motor functions, its unique role in non-motor functions has been established by various human and non-human studies^7,8,69^. Neuroimaging studies have identified two functional representations within the cerebellum: somatomotor representation in lobules I–IV and VIII and cognitive representation in the posterior cerebellum adjacent to Crus I and II^4,70,71^. GM volumes in the left Crus I and II and right lobule VIIb displayed significant volumetric correlations with the cerebral regions in the frontal and temporal lobes, which constitute the fronto-parietal and attention networks. Other cerebellar clusters, including the lobules V, VI, and X, are located at the boundaries between the somatomotor and cognitive subdivisions. The cerebellar cluster containing these regions demonstrated significant positive volumetric associations with both somatomotor cortices and higher-order association areas. Notably, clusters in lobules I–IV and VIIIa, which contain somatomotor representations, did not demonstrate a significant correlation with cerebral GM volume. While we did not directly investigate the structural or functional connectivity between the cerebrum and cerebellum, our findings align with previous research suggesting that the cerebro-cerebellar volumetric covariance is specific to the functional network topography^27,72^. This implies that the two brains are functionally interconnected through the CCTCC and share common pathogenetic mechanisms that result in dysfunction of the non-motor domain in both the cerebrum and cerebellum^27,56^.

Although the correlation results from an exploratory analysis should be interpreted with caution, this study demonstrated a trend toward associations between decreased cerebellar GM volume and impaired cognitive function in the participants with schizophrenia. The clusters associated with cognitive performance were lobules V, VI, VIIb, X, and Crus I and II, which evolved in parallel with the cerebral association areas and formed prefrontal-cerebellar loops^73^. On the other hand, clusters in lobules I–IV and VIIIa did not demonstrate any association with cognitive function. Some researchers limit the function of the cerebellum to controlling fine finger and eye movements for skilled cognitive performance^74^. However, King, et al.^75^ demonstrated that cognitive, rather than motor processes, predominantly drive cerebellar activity in the cognitive representation areas, which comprise definable functional boundaries and specialization across the cerebellum. Therefore, it is not surprising that the GM volume in lobules I–IV and VIIIa was not associated with cognitive performance in participants with schizophrenia. Taken together, understanding the cerebellum’s structural covariance with the fronto-temporo-parietal structural network in relation to cognitive function, despite the need for future research, would provide a novel insight into the pathogenesis of schizophrenia.

This study had some limitations that must be considered while interpreting the results. First, the sample size was relatively small, particularly in the CS group; therefore, our findings may have limited generalizability. However, our study highlights the importance of investigating the role of the cerebellum in the pathogenesis of schizophrenia with a focus on cerebro-cerebellar networks. Second, we could not determine the direction of causality of the GM volumetric abnormalities in the cerebrum and cerebellum using a cross-sectional design. Therefore, longitudinal studies with larger sample sizes are necessary to determine the neurobiological trajectory of schizophrenia. Third, we could not fully exclude the early effect of antipsychotic medication on GM volume since some participants with schizophrenia underwent MRI scans after the initiation of treatment. Although the duration of antipsychotic exposure before the MRI scans in this study was brief (mean = 5.2 ± 4.1 days), some reports have shown that prolonged antipsychotic treatment leads to brain volume reduction^76–78^. However, since it is unethical to keep patients off antipsychotics for research purposes, efforts were made to use the minimum dose of antipsychotics necessary to obtain voluntary cooperation from participants with schizophrenia. Finally, more comprehensive assessments of cognitive function, including social cognition, with control data will be needed to understand the non-motor role of the cerebellum.

In conclusion, our study corroborates previous findings on the involvement of the cerebellum in schizophrenia and provides compelling evidence of structural abnormalities in the cerebellar GM, particularly in the posterior lobe, in patients with schizophrenia. A smaller cerebellar GM volume observed in both the ROS and CS groups suggests that cerebellar pathogenesis may underlie the development of the illness from the early stages. Furthermore, cerebro-cerebellar GM volumetric correlations within the fronto-temporal networks demonstrated that these brain regions are closely interconnected, forming the structural basis of higher-order associative function in patients with schizophrenia. Therefore, we propose integrating the cerebellum into the current conceptual framework of schizophrenia to better understand its pathogenesis and identify novel treatment targets in relation to cerebro-cerebellar networks in schizophrenia.

### Supplementary information

Supplementary material

## Data Availability

The data supporting the findings of this study are not publicly available due to ethical restrictions for protecting participants’ confidentiality and privacy but are accessible from the corresponding author on reasonable request with the approval of the Institutional Review Board of CHA Bundang Medical Center.
