# Supplementary material for: Cerebro-cerebellar gray matter abnormalities associated with cognitive impairment in patients with recent-onset and chronic schizophrenia
Source: Schizophrenia (Heidelb). 2024 Jan 27;10(1):11. doi: 10.1038/s41537-024-00434-8 (PMC10851702; doi:10.1038/s41537-024-00434-8)
Supplement: Supplementary file 1 — Supplementary material [file 41537_2024_434_MOESM1_ESM.docx]

**SUPPLEMENTARY MATERIAL**

***S1. Cognitive Assessments***

Cognitive function was evaluated by a certified clinical psychologist, using a comprehensive neurocognitive battery. The battery consists of the similarities, comprehension, and digit span subtests of the Korean Wechsler Adult Intelligence Scale-IV (K-WAIS-IV)^1,2^, Trail-Making Test (TMT) parts A and B^3^, verbal fluency test^4^, and Rey–Kim memory test^5^. Similarities and comprehension are the measures of executive function for verbal reasoning and problem solving^6^, and forward and backward digit spans are the measures of attention and working memory^7^. The TMT-A and -B assess the information processing speed, cognitive flexibility, and visuomotor skills^8^. The verbal fluency test, consisting of letter-guided and category-guided trials, measures verbal executive control^9^. The Rey-Kim memory test is the Korean standardized version of the auditory verbal learning test (AVLT) and complex figure test (CFT), used to assess verbal and visual memory functions^10,11^.

***S2. Details on Exploratory Analysis for*** ***Cognitive Performance and Its Correlations with Cerebro-Cerebellar Gray Matter Volumes in Participants with Schizophrenia***

The extracted mean gray matter (GM) volumes of the cerebellar clusters from 2 to 5, which demonstrated significant volumetric associations with fronto-temporo-parietal regions, were positively correlated with cognitive performance across various domains, including executive function and verbal memory (Supplementary Table S2). The mean GM volume of Cluster 3, which is located across the left Crus I and II, displayed positive correlations with executive function measured by the similarities (*r* = 0.191, *p* = 0.046) and comprehension (*r* = 0.197, *p* = 0.039) subtests of the K-WAIS-IV and letter fluency test (*r* = 0.203, *p* = 0.033) and verbal memory function measured by free recall 1 to 5 of the AVLT (*r* = 0.199, *p* = 0.037). The greater GM volume in Cluster 4 was associated with higher performance in the comprehension subtest (*r* = 0.189, *p* = 0.048), letter fluency test (*r* = 0.196, *p* = 0.040), AVLT-free recall 1 to 5 (*r* = 0.211, *p* = 0.027), and AVLT-delayed recognition (*r* = 0.225, *p* = 0.018). The mean GM volume of Cluster 5, located across the left lobules V and VI, was positively correlated with the letter fluency test (*r* = 0.208, *p* = 0.029) and CFT-visual copy (*r* = 0.189, *p* = 0.048) scores. The mean GM volume of Cluster 2 positively correlated with working memory performance, as measured by the backward digit span (*r* = 0.212, *p* = 0.026).

Greater mean GM volume extracted from the cerebral regions associated with cerebellar Clusters 3 to 5 was significantly correlated with better performance on cognitive tests for executive function and verbal and visual memory (Supplementary Table S3). The GM volumes of the fronto-temporal and insular cortices associated with Cluster 3 were positively correlated with the similarities subtests, letter fluency test, and AVLT-free recall 1 to 5. The GM volumes of the insula and pars orbitalis, displaying associations with Cluster 4, were correlated with letter fluency. Greater GM volume in the fronto-temporal and insular regions related to Cluster 5 was associated with higher performance in letter fluency and CFT-visual copy.

| **Supplemantary Table S1.** Differences in cognitive function between participants with ROS and those with CS^a^ | | | | |
| --- | --- | --- | --- | --- |
|  | ROS  (*n* = 72) | CS  (*n* = 43) | *F* | *p* |
| K-WAIS-IV subtests |  |  |  |  |
| Similarities | 15.9 ± 4.7 | 13.5 ± 5.4 | 0.96 | 0.330 |
| Comprehension | 17.1 ± 6.8 | 15.1 ± 7.0 | 0.98 | 0.324 |
| Digit span - forward | 7.2 ± 1.5 | 6.5 ± 1.3 | 3.82 | 0.053 |
| Digit span - backward | 4.4 ± 1.5 | 4.4 ± 1.6 | 0.53 | 0.470 |
| Trail-making test |  |  |  |  |
| Part A | 42.5 ± 35.4 | 58.1 ± 48.8 | 2.83 | 0.095 |
| Part B | 116.2 ± 69.1 | 156.4 ± 115.3 | 1.07 | 0.302 |
| Verbal fluency test |  |  |  |  |
| Letter fluency | 31.0 ± 13.0 | 26.1 ± 14.7 | 0.01 | 0.920 |
| Category fluency | 33.7 ± 9.5 | 31.7 ± 10.0 | 0.06 | 0.811 |
| Auditory verbal learning test |  |  |  |  |
| Free recall 1–5 | 39.8 ± 11.5 | 34.1 ± 11.4 | 2.21 | 0.140 |
| Delayed recall | 7.4 ± 3.5 | 6.7 ± 3.5 | 0.16 | 0.693 |
| Delayed recognition | 12.7 ± 2.0 | 11.7 ± 2.9 | 10.69 | 0.001 |
| Complex figure test |  |  |  |  |
| Copy | 31.7 ± 6.1 | 30.5 ± 5.7 | 0.13 | 0.719 |
| Immediate recall | 16.3 ± 7.0 | 14.9 ± 11.9 | 0.80 | 0.375 |
| Delayed recall | 15.1 ± 7.2 | 13.7 ± 10.1 | 0.02 | 0.887 |
| ^a^ Age, sex, intracranial volume, duration of psychosis, and chlorpromazine equivalent dose of antipsychotics were controlled as covariates.  ROS, recent-onset schizophrenia; CS, chronic schizophrenia; K-WAIS-IV, Korean Wechsler Adult Intelligence Scale, Fourth Edition. | | | | |

| **Supplemantary Table S2.** Correlations between the cerebellar GM volumes and cognitive performance in participants with schizophrenia^a^ | | | | | | |
| --- | --- | --- | --- | --- | --- | --- |
|  | Cluster 1 | Cluster 2 | Cluster 3 | Cluster 4 | Cluster 5 | Cluster 6 |
| K-WAIS-IV subtests |  |  |  |  |  |  |
| Similarities | *r* = -0.011  *p* = 0.912 | *r* = 0.176  *p* = 0.066 | *r* = 0.191  *p* = 0.046^*^ | *r* = 0.157  *p* = 0.102 | *r* = 0.044  *p* = 0.645 | *r* = 0.090  *p* = 0.350 |
| Comprehension | *r* = -0.033  *p* = 0.731 | *r* = 0.113  *p* = 0.239 | *r* = 0.197  *p* = 0.039^*^ | *r* = 0.189  *p* = 0.048^*^ | *r* = 0.032  *p* = 0.739 | *r* = 0.131  *p* = 0.174 |
| Digit span - forward | *r* = 0.172  *p* = 0.072 | *r* = 0.182  *p* = 0.057 | *r* = 0.170  *p* = 0.075 | *r* = 0.114  *p* = 0.236 | *r* = 0.053  *p* = 0.579 | *r* = 0.105  *p* = 0.274 |
| Digit span - backward | *r* = 0.086  *p* = 0.371 | *r* = 0.212  *p* = 0.026^*^ | *r* = 0.177  *p* = 0.064 | *r* = 0.122  *p* = 0.202 | *r* = 0.089  *p* = 0.355 | *r* = 0.007  *p* = 0.946 |
| Trail-making test |  |  |  |  |  |  |
| Part A | *r* = -0.057  *p* = 0.556 | *r* = -0.100  *p* = 0.300 | *r* = -0.122  *p* = 0.205 | *r* = -0.149  *p* = 0.119 | *r* = -0.039  *p* = 0.682 | *r* = 0.005  *p* = 0.958 |
| Part B | *r* = -0.011  *p* = 0.910 | *r* = -0.055  *p* = 0.565 | *r* = -0.079  *p* = 0.413 | *r* = -0.042  *p* = 0.661 | *r* = 0.001  *p* = 0.991 | *r* = -0.107  *p* = 0.265 |
| Verbal fluency test |  |  |  |  |  |  |
| Letter fluency | *r* = 0.110  *p* = 0.255 | *r* = 0.064  *p* = 0.508 | *r* = 0.203  *p* = 0.033^*^ | *r* = 0.196  *p* = 0.040^*^ | *r* = 0.208  *p* = 0.029^*^ | *r* = 0.177  *p* = 0.065 |
| Category fluency | *r* = -0.029  *p* = 0.762 | *r* = 0.118  *p* = 0.222 | *r* = 0.178  *p* = 0.063 | *r* = 0.162  *p* = 0.090 | *r* = 0.094  *p* = 0.330 | *r* = 0.154  *p* = 0.109 |
| Auditory verbal learning test |  |  |  |  |  |  |
| Free recall 1–5 | *r* = 0.105  *p* = 0.275 | *r* = 0.103  *p* = 0.283 | *r* = 0.199  *p* = 0.037^*^ | *r* = 0.211  *p* = 0.027^*^ | *r* = 0.106  *p* = 0.268 | *r* = 0.099  *p* = 0.302 |
| Delayed recall | *r* = 0.130  *p* = 0.177 | *r* = 0.060  *p* = 0.530 | *r* = 0.175  *p* = 0.067 | *r* = 0.119  *p* = 0.215 | *r* = 0.126  *p* = 0.191 | *r* = 0.068  *p* = 0.480 |
| Delayed recognition | *r* = 0.128  *p* = 0.184 | *r* = 0.052  *p* = 0.587 | *r* = 0.097  *p* = 0.313 | *r* = 0.225  *p* = 0.018^*^ | *r* = 0.202  *p* = 0.834 | *r* = 0.076  *p* = 0.430 |
| Complex figure test |  |  |  |  |  |  |
| Copy | *r* = 0.112  *p* = 0.244 | *r* = 0.097  *p* = 0.312 | *r* = 0.157  *p* = 0.100 | *r* = 0.167  *p* = 0.081 | *r* = 0.189  *p* = 0.048^*^ | *r* = 0.159  *p* = 0.097 |
| Immediate recall | *r* = 0.119  *p* = 0.215 | *r* = -0.008  *p* = 0.937 | *r* = 0.118  *p* = 0.219 | *r* = 0.141  *p* = 0.142 | *r* = 0.071  *p* = 0.462 | *r* = 0.187  *p* = 0.050 |
| Delayed recall | *r* = 0.072  *p* = 0.455 | *r* = -0.027  *p* = 0.781 | *r* = 0.053  *p* = 0.584 | *r* = 0.090  *p* = 0.350 | *r* = 0.054  *p* = 0.578 | *r* = 0.187  *p* = 0.051 |
| ^a^ Age, sex, intracranial volume, duration of psychosis, and chlorpromazine equivalent dose of antipsychotics were controlled as covariates.  ^*^ *p* < 0.05 (uncorrected).  GM, gray matter; K-WAIS-IV, Korean Wechsler Adult Intelligence Scale, Fourth Edition. | | | | | | |

| Supplementary Table S3. Cerebral gray matter volume correlations with the cerebellar clusters and cognitive function in participants with schizophrenia | | |
| --- | --- | --- |
| Cerebellar  cluster | Cerebral region | Correlation with cognitive function^a, b^ |
|  |  |  |
| Cluster 1 | None |  |
| Cluster 2 | Right |  |
|  | Superior parietal gyrus |  |
| Cluster 3 | Left |  |
|  | Precentral gyrus | Similarities: *r* = 0.222, *p* = 0.020 |
|  |  | Letter fluency: *r* = 0.290, *p* = 0.002 |
|  | Superior frontal gyrus | Letter fluency: *r* = 0.234, *p* = 0.014 |
|  |  | Letter fluency: *r* = 0.200, *p* = 0.036 |
|  | Superior temporal gyrus | Letter fluency: *r* = 0.230, *p* = 0.016 |
|  | Right |  |
|  | Fusiform gyrus |  |
|  | Pars orbitalis | Letter fluency: *r* = 0.241, *p* = 0.011 |
|  | Insula | Letter fluency: *r* = 0.276, *p* = 0.003  Verbal free recall 1–5: *r* = 0.219, *p* = 0.021 |
|  | Superior temporal gyrus |  |
| Cluster 4 | Left |  |
|  | Insula | Letter fluency: *r* = 0.198, *p* = 0.038 |
|  | Frontal pole |  |
|  | Right |  |
|  | Transverse temporal gyrus |  |
|  | Pars orbitalis | Letter fluency: *r* = 0.225, *p* = 0.018 |
| Cluster 5 | Left |  |
|  | Superior temporal gyrus |  |
|  | Rostral anterior cingulate gyrus | Letter fluency: *r* = 0.191, *p* = 0.045 |
|  |  | Visual copy: *r* = 0.244, *p* = 0.010 |
|  | Precentral gyrus | Letter fluency: *r* = 0.236, *p* = 0.013 |
|  |  | Visual copy: *r* = 0.250, *p* = 0.008 |
|  | Insula | Letter fluency: *r* = 0.219, *p* = 0.022 |
|  | Lateral occipital gyrus |  |
|  | Superior parietal gyrus |  |
|  | Right |  |
|  | Transverse temporal gyrus | Letter fluency: *r* = 0.210, *p* = 0.028  Visual copy: *r* = 0.208, *p* = 0.029 |
|  | Superior temporal gyrus | Letter fluency: *r* = 0.191, *p* = 0.046 |
|  | Middle temporal gyrus |  |
| Cluster 6 | None |  |
| ^a^  Age, sex, intracranial volume, duration of psychosis, and chlorpromazine equivalent dose of antipsychotics were controlled as covariates.  ^b^ Only cognitive tests that showed significant associations with each cerebellar cluster were included in the correlation analysis. | | |

**REFERENCES**

1 Wechsler, D. *Wechsler Adult Intelligence Scale (4th ed.)*. (Pearson Assessment, San Antonio, TX, 2008)

2 Hwang, S., Kim, J., Park, K., Chey, J. & Hong, S. *Korean Wechsler Adult Intelligence Scale-IV*. (Korean Psychology Corporation, Daegu, 2012)

3 Reitan, R. M. Validity of the Trail Making Test as an indicator of organic brain damage. *Perceptual and motor skills* **8**: 271-276 (1958).

4 Thurstone, L. L. *Primary mental abilities*. (University of Chicago Press, Chicago, IL, 1938)

5 Kim, H. K. Assessment of memory disorders using Rey-Kim memory test. *Korean J Rehabil Psychol* **8**: 29-48 (2001).

6 Higginson, C. I., King, D. S., Levine, D., Wheelock, V. L., Khamphay, N. O. & Sigvardt, K. A. The relationship between executive function and verbal memory in Parkinson's disease. *Brain Cogn* **52**: 343-352 (2003).

7 Conklin, H. M., Curtis, C. E., Katsanis, J. & Iacono, W. G. Verbal working memory impairment in schizophrenia patients and their first-degree relatives: evidence from the digit span task. *Am J Psychiatry* **157**: 275-277 (2000).

8 Bowie, C. R. & Harvey, P. D. Administration and interpretation of the Trail Making Test. *Nat Protoc* **1**: 2277-2281 (2006).

9 Troyer, A. K., Moscovitch, M. & Winocur, G. Clustering and switching as two components of verbal fluency: evidence from younger and older healthy adults. *Neuropsychology* **11**: 138-146 (1997).

10 Rey, A. L'examen psychologique dans les cas d'encéphalopathie traumatique. (Les problems.). [The psychological examination in cases of traumatic encepholopathy. Problems.]. *Arch Psychol* **28**: 215-285 (1941).

11 Rey, A. *L'examen clinique en psychologie*. (Presses universitaires de France, Paris, 1964)
